# X-derived marker chromosome in patient with mosaic Turner syndrome and Dandy-Walker syndrome: a case report

**DOI:** 10.1186/s13039-017-0344-2

**Published:** 2017-11-17

**Authors:** Alena S. Telepova, Svetlana A. Romanenko, Natalya A. Lemskaya, Yulia V. Maksimova, Asia R. Shorina, Dmitry V. Yudkin

**Affiliations:** 1 Institute of Molecular and Cellular Biology SB RAS, Lavrentieva ave. 8/2, Novosibirsk, 630090 Russia; 2 Novosibirsk State University, Novosibirsk, 630090 Russia; 3 Novosibirsk State Medical University, Novosibirsk, 630091 Russia; 4 Novosibirsk City Clinical Hospital No.1, Novosibirsk, 630047 Russia

**Keywords:** Marker chromosome, Turner syndrome, Dandy-Walker syndrome, X-chromosome

## Abstract

**Background:**

Small supernumerary marker chromosomes can be derived from autosomes and sex chromosomes and can accompany chromosome pathologies, such as Turner syndrome.

**Case presentation:**

Here, we present a case report of a patient with mosaic Turner syndrome and Dandy-Walker syndrome carrying a marker chromosome. We showed the presence of the marker chromosome in 33.8% of blood cells. FISH of the probe derived from the marker chromosome by microdissection revealed that it originated from the centromeric region of chromosome X. Additionally, we showed no telomeric sequences and no XIST sequence in the marker chromosome. This is the first report of these two syndromes accompanied by the presence of a marker chromosome.

**Conclusion:**

Marker chromosome was X-derived and originated from centromeric region. Patient has mild symptoms but there is no XIST gene in marker chromosome.

**Trial registration:**

CPG137. Registered 03 March 2017.

## Background

Small supernumerary marker chromosomes (sSMC) are structurally abnormal chromosomes. The size of sSMC is smaller than chromosome 20 on metaphase spread [1]. sSMC can be derived from autosomes and sex chromosomes. Occasionally, sSMC can accompany chromosome pathologies, such as Turner syndrome [2]. Turner syndrome is a genetic disorder caused by karyotype 45,X with or without mosaicism [3]. Described by Henry Turner in 1938, it consists of a constellation of phenotypic findings: short stature, sexual infantilism, webbed neck, and cubitus valgus. Usually, sSMC in Turner syndrome (sSMC^T^) are derived from sex chromosomes [4]. Most sSMC^T^(X) form ring-chromosomes, but rare sSMC^T^(Y) are inverted duplicated/isodicentric ones. The frequency of sSMC^T^ is 1:100,000 in a population [3]. The phenotypic variability of these mosaics is largely dependent on the size of the marker chromosome and the presence of a functioning XIST gene in the X-inactivation center (Xq13). If the marker chromosome undergoes inactivation, patients with sSMC^T^ have a mild Turner variant phenotype [5].

In this case report we described an exceptional patient with both Turner and Dandy-Walker syndromes carrying a sSMC^T^ derived from the centromere region of the X chromosome. The combination of a Dandy-Walker syndrome and sSMC^T^ was described for the first time.

## Materials and methods

### Clinical study

The clinical study of the patient included consultations with medical specialists such as clinical psychologists, neurologists, speech therapists and MRI research.

### Metaphase preparation and GTG banding

Metaphase chromosome preparations were obtained from lymphocyte cultures according to standard procedures [6]. GTG banding was carried out as described previously [7]. Metaphase spreads were analyzed using an Olympus BX 53 microscope and the “VideoTest Karyo 3.1” (Zenit, Russia) software.

### Microdissection and FISH

Marker chromosomes were dissected as described earlier [8] using an Olympus IX 51 microscope and the micromanipulator of Eppendorf Transferman NK2. DNA of microdissected chromosomes was amplified by GenomePlex Complete Whole Genome Amplification (WGA) Kit (Sigma-Aldrich, USA). Probes were labeled with digoxigenin. Every library was obtained from one copy of marker chromosome.

FISH of microdissected probes, sorted human chromosome probes and telomeric probes was carried out as described [8]. Slides were analyzed using an Olympus BX 53 fluorescence microscope and the “VideoTest FISH 2.0” (Zenit, Russia) software.

### PCR screening

PCR screening of painting probes was carried out with primers: XISTF 5’AGTGTACCTACCGCTTTGGC3’ and XISTR 5’TCCTCTGCCTGACCTGCTAT3’ (ref. seq. NG_016172.1) for XIST, CHXF 5’TTCTCTGTCCTGCGACCTTG3’ and CHXR 5’GCTCAAAAGACTGGGCACCT3’ (ref. seq. NC_000023.11) for centromeric region of chromosome X and CH3F 5’GTGACTTCCCAACCTGGATTCT3’ and CH3R 5’GATCATCCCAAAGGACATCAACT3’ (ref. seq. NG_047144.1) for CNTN6 gene on chromosome 3. Painting probes of microdissection-derived marker chromosomes and sorted chromosomes X and 3 were used as a template.

## Case presentation

### Clinical study

The patient is a 13-year-old female with growth retardation (nanism, 137 cm), a short neck, low hair growth on the neck, widely spaced nipples, a shield chest, secondary sexual characteristics deficiency, and the presence of episodic hyperglycemia. Her body mass index is 25. The parents of the patient have normal growth (mother – 175 cm, father – 180 cm). MRI study revealed Dandy-Walker syndrome and hydrocephaly. Hypoplasia of segment A1 of the anterior cerebral artery and segment P1 of the posterior cerebral artery were revealed.

### Karyotypic study

**Fig. 1:**
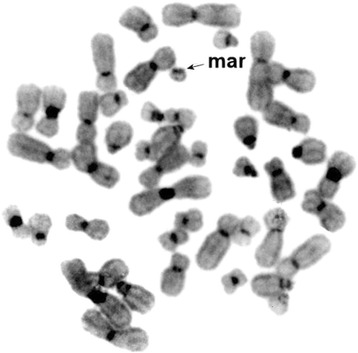
Patient’s metaphase spread, with the arrow indicating the marker chromosome

**Fig. 2:**
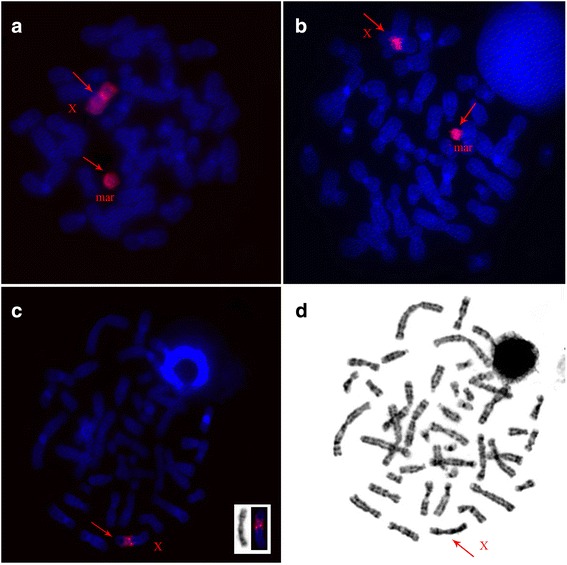
Examples of fluorescent in situ hybridization: **a** Localization of human chromosome X painting probe (red) on chromosomes of the patient. **b** Localization of microdissected probe of marker chromosome on chromosomes of the patient. **c** Localization of microdissected probe of marker chromosome on chromosomes of the healthy control after G-banding (**d**). Box on (**c**) is localization of signal on G-banded chromosome X. Arrows indicate signal localization

The patient has karyotype 45,X/46,X,+mar. The marker chromosome was found in 33.8% of the metaphase spreads (*N* = 80). It was small and comparable in size with chromosome 20. Although DAPI staining showed that this chromosome looks such as a small acrocentric chromosome, localization of the telomeric probe revealed no signals on it (Fig. 1). However, as shown previously, the absence of telomeres cannot indicate unambiguously that the chromosome is a ring [9]. FISH of the set of human whole chromosomes-specific probes showed the localization of chromosome X painting probe on the whole length of the marker chromosome (Fig. 2a). To visualize the boundaries of the derivate formation, the marker chromosome was microdissected, and the derived probe was localized on chromosomes of the patient and a healthy control. In total, we obtained 4 libraries. In the patient cells each probe painted the marker chromosome and the centromeric region of chromosome X (Fig. 2b). In the healthy control, probes painted the centromeric region of chromosome X only (Fig. 2c, d). This localization showed the marker chromosome was derived from region Xp11.23-q13.2. (Fig. 2c – box).

### PCR screening for XIST gene

**Fig. 3:**
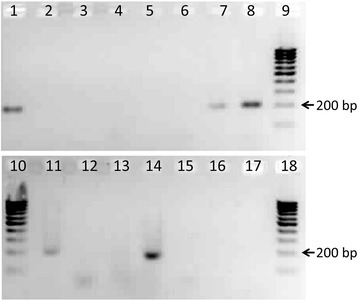
Results of PCR screening of marker chromosomes for XIST gene presence. Primers: CH3R/F on lines 1-5; CHXR/F on lines 6-8, 11, 12; XISTF/R on lines 13-17. DNA templates: chromosome 3 painting probe on lines 1, 6, 13; chromosome X painting probe on lines 2, 7, 14; microdissection-derived marker chromosome (mar1) on lines 3, 8, 15; microdissection-derived marker chromosome (mar2) on lines 4, 11, 16; negative controls on lines 5, 12, 17; 100 bp ladder on lines 9, 10, 18

To test whether XIST gene presents in marker chromosome or not we made PCR screening of two separate copies of microdissection-derived probes of the marker chromosome (mar1 and mar2) and painting probes of chromosomes X and 3 as controls (see Materials and Methods). Both analyzed marker chromosomes do not contain XIST sequences whereas X centromeric sequences are presented in marker chromosomes painting probes (Fig. 3).

## Discussion

In 2014, Mazzaschi R. et al. described a female patient with sSMC^T^ derived from the centromere region of the chromosome X. In that case, the proband had poor growth, global developmental delay, type I diabetes, clonic seizures, cardiomyopathy, hepatic adenomas, and skeletal dysplasia. Moreover, she had delayed secondary sexual characteristic development and ovarian failure and growth hormone deficiency [5]. The clinical symptoms of the patient in our research differed from those described above. The manifestations of Turner’s syndrome in our case were milder; the patient had not expressed cognitive failure, clonic seizures or damage to other organs. However, she had Dandy-Walker syndrome. Dandy-Walker syndrome is defined by hypoplasia of the cerebellar vermis and dilation of the fourth ventricle [10]. Patients with this syndrome often have motor deficits such as delayed motor development, hypotonia and ataxia; further, approximately half have mental retardation and some have hydrocephalus [11]. Based on the results of an MRI study of the brain, hydrocephalus was diagnosed in this case as well.

A female patient with symptoms similar to the case was described earlier. The ten-year-old girl had typical manifestations of Turner’s syndrome – short stature, webbing of neck, cubitus valgus, shield chest, congenital dislocation of hip, renal anomalies, clinodactyly, unilateral simian crease on right palm, acyanotic congenital heart disease and small patent ductus arteriosus. However, intellectual insufficiency did not occur in that patient, and further analysis revealed that the marker chromosome in her karyotype was derived from chromosome 14 [2].

Combining the conventional karyotyping and FISH data allowed us to characterize the genetic content of the marker chromosome. The derivate corresponds to Xp11.23-q13.2 region, which contains the gene XIST. This gene is responsible for the X chromosome inactivation, which is necessary to realize the epigenetic mechanism of dosage compensation. The structurally abnormal chromosome X without XIST could not be inactivated, which led to an increase in the dose of genes and, consequently, to the appearance of features of Turner’s syndrome. This, in turn, led to a more severe phenotype of Turner’s syndrome [5]. In the case report we found no XIST gene in X derived marker chromosome that means other reason of mild symptoms.

## Conclusion

In this clinical case we described the patient with a mild phenotype of Turner’s syndrome. The proband does not have intellectual disability or severe diseases of internal organs in absence of XIST gene in X-derived marker chromosome.
